# The Relationship between Serum Zonulin Level and Clinical and Laboratory Parameters of Childhood Obesity

**DOI:** 10.4274/jcrpe.3682

**Published:** 2017-03-01

**Authors:** Tuncay Küme, Sezer Acar, Hale Tuhan, Gönül Çatlı, Ahmet Anık, Özlem Gürsoy Çalan, Ece Böber, Ayhan Abacı

**Affiliations:** 1 Dokuz Eylül University Faculty of Medicine, Department of Medical Biochemistry, İzmir, Turkey; 2 Dokuz Eylül University Faculty of Medicine, Department of Pediatric Endocrinology, İzmir, Turkey

**Keywords:** Tight junctions, zonulin, insulin resistance, obesity, childhood

## Abstract

**Objective:**

To investigate the relationship between zonulin levels and clinical and laboratory parameters of childhood obesity.

**Methods:**

The study included obese children with a body mass index (BMI) >95^th^ percentile and healthy children who were of similar age and gender distribution. Clinical (BMI, waist circumferences, mid-arm circumference, triceps skinfold, percentage of body fat, systolic blood pressure, diastolic blood pressure) and biochemical (glucose, insulin, lipid levels, thyroid function tests, cortisol, zonulin and leptin levels) parameters were measured.

**Results:**

A total of 43 obese subjects (23 males, mean age: 11.1±3.1 years) and 37 healthy subjects (18 males, mean age: 11.5±3.5 years) were included in this study. Obese children had significantly higher insulin, homeostasis model assessment of insulin resistance, triglyceride, total cholesterol, low-density lipoprotein cholesterol, high-density lipoprotein cholesterol (HDL-C), zonulin and leptin levels than healthy children (p<0.05), while glucose levels were not different (p>0.05). Comparison of the obese children with and without insulin resistance showed no statistically significant differences for zonulin levels (p>0.05). Zonulin levels were found to negatively correlate with HDL-C and positively correlate with leptin levels, after adjusting for age and BMI.

**Conclusion:**

To the best of our knowledge, this is the first study investigating the relationship between circulating zonulin level (as a marker of intestinal permeability) and insulin resistance and leptin (as markers of metabolic disturbances associated with obesity) in childhood obesity. The results showed that zonulin was significantly higher in obese children when compared to healthy children, a finding indicating a potential role of zonulin in the etiopathogenesis of obesity and related disturbances.

**WHAT IS ALREADY KNOWN ON THIS TOPIC?**

Zonulin is a protein that increases the intestinal permeability in small intestinal system by modulating the intracellular tight junctions.

**WHAT THIS STUDY ADDS?**

The present study is the first study to compare the serum zonulin levels between obese and non-obese children. We demonstrated that serum zonulin levels were higher in obese children when compared to healthy children, which may play a role in the pathogenesis of obesity and related metabolic disturbances.

## INTRODUCTION

The prevalence of an overweight state, which is an important cause of morbidity and mortality in the world, has increased to pandemic proportions among children and adolescents. Imbalance between energy intake and expenditure as well as sedentary lifestyle are the leading causes of obesity (1). Obesity is associated with systemic microinflammation due to the release of proinflammatory peptides in visceral adipose tissue. Systemic microinflammation in obesity is the major cause in the pathogenesis of metabolic disorders such as insulin resistance (IR), dyslipidemia, type 2 diabetes (2). Recent evidence suggests a possible role of intestinal barrier dysfunction from releasing proinflammatory peptides in obesity (3).

The intestines have a function as a barrier to protect the body from infectious, toxic, allergic agents as well as from antigenic loads and also from caloric loads. Recent studies which report an increase in intestinal permeability and absorption together with a decrease in intestinal motility in patients with metabolic disorders indicate that there is a link between intestinal barrier dysfunction and metabolic disorders (3).

The structure, function, and regulation of interepithelial zonula occludens [tight junction (TJ)] are important for intestinal barrier function. Firstly, the zonula occludens toxin (zot), an enterotoxin secreted by *Vibrio Cholera*, was reported to affect the TJs. Subsequently, human zonulin, a physiological mediator in 47 kDa protein structure which regulates the permeability of intestinal TJ and acts as an agent of innate immunity, was isolated as a homolog of zot. Intestinal TJ dysfunction and upregulation of zonulin were found to be the primary defects in these diseases (4). Circulating zonulin levels are considered to be a useful marker of intestinal permeability. Recently, higher circulating zonulin levels were reported in obese adult subjects as compared to the non-obese and also in adults with glucose intolerance as compared to those with normal glucose tolerance (3,5). Significantly higher serum zonulin levels were also reported in obese subjects with biopsy-confirmed nonalcoholic fatty liver disease (NAFLD) than in obese subjects without NAFLD (4).

Leptin, the protein product of the *ob* gene, was shown to be involved in the regulation of food intake, energy consumption, body weight, and glucose metabolism (6). It was shown that leptin concentrations change in nutritional states such as fasting followed by a subsequent increase in food intake (7). However, there are no studies which evaluate the association between serum zonulin and leptin levels.

In the present study, we aimed to investigate the relationship of circulating zonulin level as an intestinal permeability marker and that of leptin as a metabolic disorders marker in childhood obesity.

## METHODS

Forty-three obese children with a body mass index (BMI) greater than the 95^th^ percentile according to the standards of the Centers for Disease Control and Prevention (CDC-2000) and 37 healthy children of similar age, gender, and pubertal stage distribution with a BMI between the 5^th^ and 85^th^ percentile were consecutively enrolled in the study (8). Patients and control subjects with chronic diseases (cardiovascular, gastrointestinal, and respiratory), a history of drug use (steroids and antipsychotics), endocrine pathology (Cushing syndrome and hypothyroidism), or suspected syndromes associated with obesity (Prader-Willi and Laurence-Moon-Biedl syndromes) were excluded from the study. Subjects with a recent history of upper airway infection, gastroenteritis, and use of antibiotics were also excluded.

All subjects underwent a thorough physical examination. A biochemical evaluation including thyroid function tests and serum cortisol measurement for probable endocrine pathology was performed in all obese subjects.

The study was initiated after the approval of the local ethics committee of Dokuz Eylül University Faculty of Medicine. A written informed consent of the parent(s) of each subject was also obtained before the study.

Height was measured using a Harpenden stadiometer with a sensitivity of 0.1 cm. Weight was measured using a SECA scale with a sensitivity of 0.1 kg, with all clothing removed except undergarments. BMI was calculated by dividing weight (kg) by height squared (m2). Waist circumference (WC) and mid-upper arm circumferences (MAC) were measured using standard techniques. Triceps skinfold thickness (TSF) (in millimeters) was measured with a Harpenden skinfold caliper. The percentage of body fat (PBF) was measured using bioelectric impedance analysis (Tanita BC-418, Tokyo, Japan).

Findings for pubertal development were evaluated according to Tanner staging (9). A testicular volume of ≥4 mL in males and breast development of stage 2 and over in females were considered to be findings of puberty.

Blood pressure was measured by one of the investigators using a validated protocol. Systolic blood pressure (SBP) and diastolic blood pressure (DBP) were measured twice at the right arm after a 10-min rest in the supine position using a calibrated sphygmomanometer. Hypertension was defined as blood pressure values above the 95^th^ percentile for height, age, and gender (10).

The venous blood samples were collected in plane tubes after 10-12 h of night fasting. The plane tubes were centrifuged at 1200xg for 10 minutes and serum samples were transferred into the Eppendorf tubes using plastic Pasteur pipettes. Routine parameters (glucose, insulin, lipids, thyroid function tests, cortisol) were analyzed on the same day. Samples to be analyzed for special parameters (zonulin, leptin) were stored at -80 °C until analysis.

Fasting serum glucose, triglyceride (TG), total cholesterol (TC), and high-density lipoprotein cholesterol (HDL-C) concentrations were measured enzymatically using DP Modular Systems (Roche Diagnostic Corp., Indianapolis, IN). Low-density lipoprotein cholesterol (LDL-C) levels were calculated using the Friedewald formula when plasma TGs were <400 mg/dL. Serum insulin was measured according to the electrochemiluminescence immunoassay method, using an automated immunoassay analyzer (Immulite 2500 Insulin, Diagnostic Products Corporation, Los Angeles, CA, USA). IR was evaluated according to the homeostasis model assessment of IR (HOMA-IR) index. Different cut-off values for prepubertal and pubertal subjects were determined based on 85^th^ percentile values of control cases (prepubertal >2.5, pubertal >4) (11).

Serum leptin levels (catalog no: EK0595 and EK0437, Boster Biological Technology Co Ltd, Wuhan, China) and zonulin levels (catalog no: CSB-EL028107HU and CSBEQ27649HU, CUSABIO Biotech Co Ltd, Wuhan, China) were measured by enzyme linked immunosorbent assay kit (ELISA) based on the principle of competitive enzyme immunoassay. In this assay, the microplate in the kit is pre-coated with antibody specific to the analyte. The standard is reconstituted and prepared by serial dilution with sample diluents. The serum samples are diluted 1:10 with sample diluents for leptin and adiponectin assays and 1:2000 for zonulin. Standards and samples are loaded into the appropriate microtiter plate wells and any analyte present is bound by immobilized antibody. After removing any unbound substances, a biotin-conjugated antibody specific to the analyte is added to the wells. After washing, avidin-conjugated peroxidase is added to the wells. Following a wash to remove any unbound avidin-enzyme reagent, a substrate solution is added and color develops in proportion to the amount of analyte. The color development is stopped and the intensity of the color is measured spectrophotometrically at a wavelength of 450 nm. A standard curve of known concentration of analyte is established and the concentration of analyte in the samples is calculated accordingly. The ELISA assays for leptin and zonulin had a sensitivity of <10 pg/mL and 0.156 ng/mL, a detection range of 62.5-4000 pg/mL and 0.625-40 ng/mL, an intraassay coefficient of variation (CV) of <10% and <8%, as well as interassay CV of <10% and <10%, respectively.

### Statistical Analysis

The analyses of the data were conducted with Statistical Package for the Social Sciences 16.0.1 (SPSS Inc., Chicago, IL, USA). The distribution of the data was evaluated with the Kolmogorov-Smirnov test. For numerical comparisons, the student’s t-test or Mann-Whitney U-test were used according to the distribution of the measured parameters. Categorical variables were compared using the chi-square test. A correlation analysis was performed using Spearman’s correlation analysis. Since zonulin levels were not normally distributed, they were transformed to logarithmic values for multivariate linear regression analysis. Variables with a p-value < 0.05 in the bivariate correlation analysis were included in a multivariate linear regression analysis model to assess the independent determinants of serum zonulin level. A partial correlation was also performed, with serum zonulin as a dependent variable, controlling for potential confounders such as age and BMI. The data were presented as mean ± standard deviation or median and interquartile range (IQR). In all statistical tests, p-values <0.05 were considered significant.

## RESULTS

**Figure 1:**
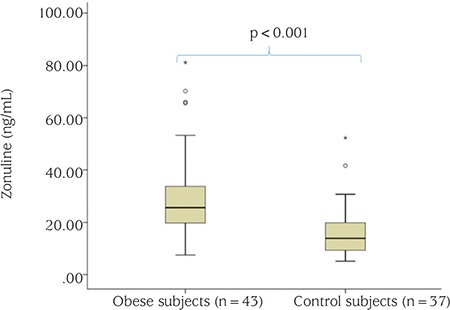
Zonulin levels in obese and healthy subjects

A total of 43 obese subjects (23 males, 20 pubertal, mean age: 11.1±3.1 years) and 37 healthy subjects (14 males, 23 pubertal, mean age: 11.5±3.6 years) were included in the study. The groups were similar for age, gender, and pubertal status. There were significant differences between obese and healthy children in terms of BMI, BMI- standard deviation score (SDS), WC, MAC, TSF, fat mass, PBF, SBP, and DBP (p<0.05), as shown in Table 1. Obese children had significantly higher insulin, HOMA-IR, TG, TC, LDL-C, HDL-C, zonulin, and leptin levels than the healthy children (p<0.05), while glucose levels were similar in the two groups (p>0.05) (Figure 1, Table 2).

Comparison of the obese children regarding their findings on IR showed statistically significant differences for BMI, MAC, WC, fat mass, PBF, insulin, and HOMA-IR (p<0.05). Age, sex, BMI-SDS, TSF, SBP, DBP, serum glucose, TG, TC, LDL-C, HDL-C, zonulin, and leptin levels were similar in the insulin resistant and non-resistant obese children (p>0.05) (Table 3).

Spearman’s correlation analysis revealed that serum zonulin levels negatively correlated with age and HDL-C, while positive correlated with BMI-SDS, PBF, LDL-C, HDL-C, and leptin levels in the entire cohort. Zonulin level were significantly associated with only HDL-C and leptin levels, after adjusting for age and BMI (r=-0.348, p=0.026; r=0.417, p=0.007, respectively) (Table 4). In the multivariate backward regression analysis (r2=0.503, p<0.001), log-transformed zonulin was significantly associated with BMI-SDS (β-coefficient 0.398, p<0.001), and HDL-C (β-coefficient -0.379, p<0.001), which explained 47.5% of the variance.

## DISCUSSION

Experimental studies have demonstrated a close association between intestinal permeability and the pathogenesis of obesity (12,13,14,15,16). The molecular transport between the intestinal lumen and the submucosa is regulated by dynamic TJ structures found between the intestinal cells (17). The recently described peptide, zonulin, is known to increase the intestinal permeability by altering the paracellular TJs. The weakening of the intestinal barrier leads to an increased exposure to pathogens and allergens (18,19). On the other hand, various organisms are known to have an effect on intestinal permeability and to live symbiotically in the intestinal system (20). This structure, referred to as the intestinal microbiota, is known to protect the body against various pathogens by creating a mechanic barrier on the surface mucosa of the intestinal system (20). Many studies have demonstrated that the microbiota is damaged and intestinal permeability increased in individuals exposed to a high-fat diet, leading to obesity and metabolic abnormalities (12,21). The mechanical barrier formed by the microbiota of the intestinal system is altered by changes in dietary habits and by increased exposure of intestinal cells to antigens and pathogens. Consequently, the development of inflammation in the intestinal microbiota is suggested to increase zonulin expression (22,23,24). Furthermore, adipokines and cytokines [tumor necrosis factor-α (TNF-α), interleukin-6 (IL-6), and transforming growth factor-β], induced by fat tissues in obese individuals, trigger a metabolic imbalance (IR, type 2 diabetes mellitus, etc.) by causing subclinical systemic inflammation (25). Markers of subclinical inflammation, particularly IL-6, have been suggested to regulate the gene expression of haptoglobulin-2 (HP-2) known to encode zonulin protein (3,26). In conclusion, zonulin expression is thought to be regulated by subclinical systemic inflammation as well as by local intestinal inflammation.

Increased serum zonulin levels, by weakening the cytoskeletal structure between intestinal cells, are suggested to lead to IR and to also affect the other aspects of the metabolic syndrome in obese individuals through an increase in mucosal absorption surface (5). Studies investigating the relationship between zonulin, which has an effect on increased intestinal permeability, and IR, have yielded varying results (3,5,27). Moreno-Navarrete et al (3) were the first to investigate the association between zonulin and IR and demonstrated that serum zonulin levels were significantly higher in obese individuals when compared to non-obese individuals. In the same study, serum zonulin levels were found to correlate with anthropometric (BMI and waist-hip ratio), metabolic (fasting TG levels, uric acid, HDL-C levels, and insulin sensitivity index) and inflammatory parameters (IL-6). On the other hand, multi-regression analyses have shown that the fundamental relationship of zonulin levels is with IR index and have also demonstrated that this relationship is made possible by the subclinical inflammatory marker IL-6. In a study conducted by Zhang et al (5) on three adult patient groups with normal glucose tolerance, impaired glucose tolerance, and type 2 diabetes mellitus, zonulin was found to have a positive correlation with IR. However, Zak-Golab et al (27) could not demonstrate any relationship between zonulin levels and IR in obese adult patients and interpreted their results to be due to the small sample size and also to their inability to use oral glucose tolerance test (OGTT) instead of the HOMA-IR index in the evaluation of IR. Zonulin, which is considered a marker of intestinal permeability, is suggested to play a role in the development of the metabolic syndrome (IR, type 2 diabetes mellitus, and dyslipidemia) found in obese individuals. Similar to the findings reported by Moreno-Navarrete et al (3), our study demonstrated that serum zonulin levels were higher in obese children when compared to healthy children. This result suggests that zonulin, which is known to increase intestinal permeability, may play a role in the pathogenesis of obesity and related metabolic disturbances. Our findings, similar to those reported by Zak-Golab et al (27), also failed to show any relationship between serum zonulin levels and the IR index, HOMA-IR. However, this finding, namely, the absence of a relationship with IR index, may also be attributed to the small sample size in our study and also to the fact that metabolic syndrome was not as prominent in children as in adults.

Patients with IR have been reported to have a statistically significantly high level of TC and a low HDL-C level (28). Zhang et al (5) demonstrated that serum zonulin levels and IR have a positive correlation with TG and TC, but a negative correlation with HDL-C. In this study, zonulin was suggested to cause an increase in adipose tissue through the endocannabinoid pathway by increasing intestinal permeability, thereby leading to the development of dyslipidemia (5). However, in our study, serum zonulin levels and IR were not shown to have a relationship with LDL-C, TG, and TC, but were shown to have a negative correlation with HDL-C, which is in line with previous reports (5,28). Although the mechanism of the relationship of serum zonulin with HDL-C is not well understood, it is thought to be a precursor in the development of dyslipidemia and cardiometabolic imbalance.

Leptin is secreted by adipose tissue and its levels increase with the amount of adipose tissue (29). Zonulin is a peptide that increases intestinal permeability by altering the structure of TJs and is suggested to have a potential role in the etiopathogenesis of obesity (3). Hence, a close relationship between zonulin and leptin levels might be expected. However, to the best of our knowledge, there are no studies investigating the correlation between serum zonulin concentrations and serum leptin levels in children. In the present study, we found a significant positive correlation between serum zonulin concentrations and leptin levels in obese individuals.

Some limitations need to be acknowledged regarding the present study. Firstly, inflammatory markers (IL-6, TNF-α,C-reactive protein, etc.) were not measured and hence their relationship with serum zonulin could not be evaluated due to financial constraints. Moreover, the evaluation of IR was made using the HOMA-IR index, instead of the more sensitive OGGT. Although OGTT is known to be a more sensitive index than HOMA-IR in the evaluation of IR, a strong correlation was demonstrated between IR indices detected by HOMA-IR and OGTT (30). Another point is the relationship between serum zonulin levels and celiac disease. It has been claimed that celiac disease may lead to obesity by increasing serum zonulin levels (31). Our study did not include evaluation of the obese children in terms of celiac disease.

In conclusion, the results of the present study showed that zonulin and leptin were significantly higher in obese children when compared to healthy children. Furthermore, it was reported for the first time in obese children that zonulin did not correlate with any of the anthropometric and metabolic parameters, except with serum HDL-C levels. However, further studies identifying the zonulin receptor and/or other possible cofactors will be required to elucidate the exact role of zonulin in obesity and/or IR.

## Figures and Tables

**Table 1:**
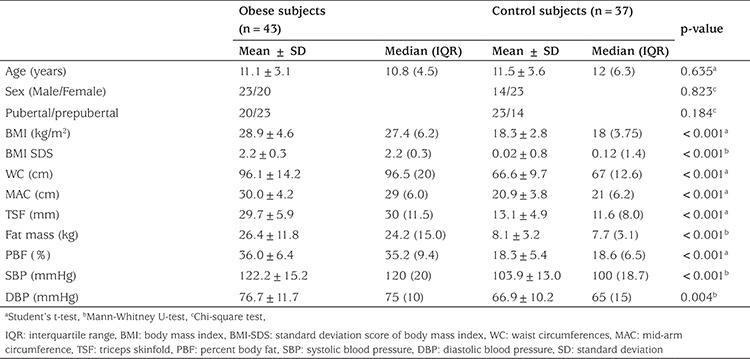
Clinical characteristics in the obese and control groups

**Table 2:**
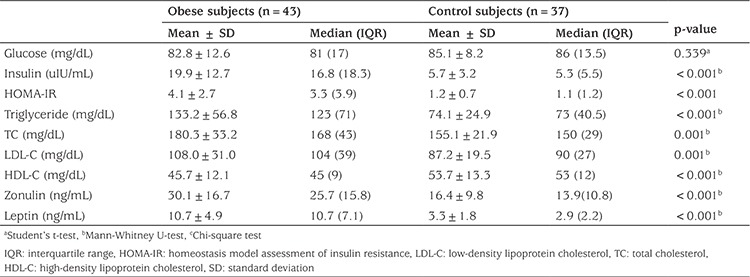
Laboratory findings in the obese and control groups

**Table 3:**
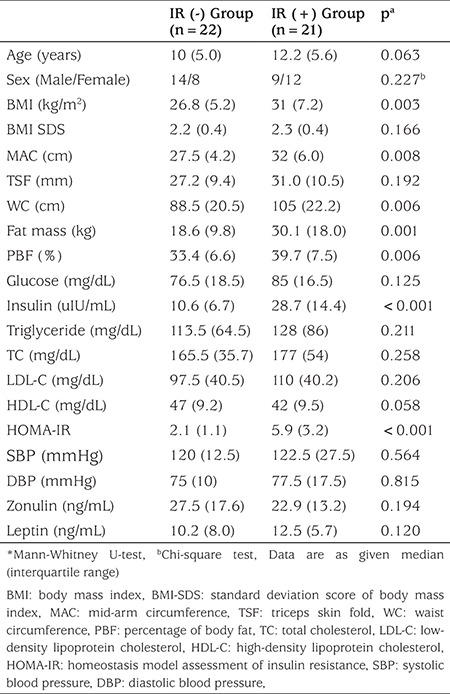
Clinical and laboratory findings in obese patients with and without insulin resistance

**Table 4:**
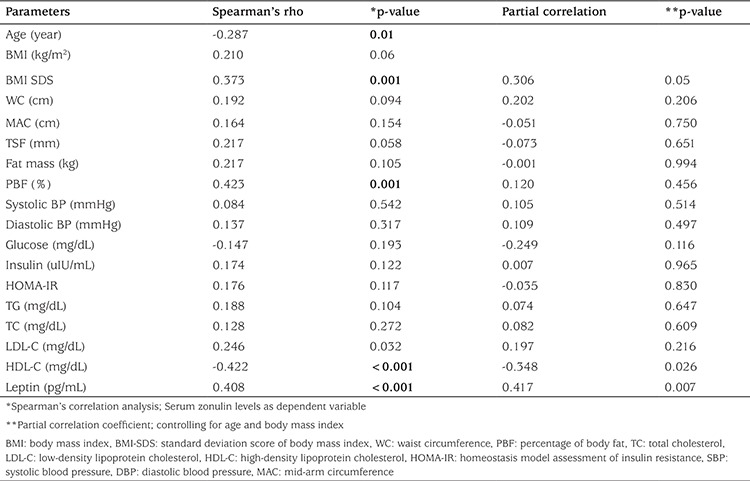
Correlation coefficients and partial correlation coefficients between zonulin levels and anthropometrics and laboratory parameters

## References

[1] Ng M, Fleming T, Robinson M, Thomson B, Graetz N, Margono C, Mullany EC, Biryukov S, Abbafati C, Abera SF, Abraham JP, Abu-Rmeileh NM, Achoki T, AlBuhairan FS, Alemu ZA, Alfonso R, Ali MK, Ali R, Guzman NA, Ammar W, Anwari P, Banerjee A, Barquera S, Basu S, Bennett DA, Bhutta Z, Blore J, Cabral N, Nonato IC, Chang JC, Chowdhury R, Courville KJ, Criqui MH, Cundiff DK, Dabhadkar KC, Dandona L, Davis A, Dayama A, Dharmaratne SD, Ding EL, Durrani AM, Esteghamati A, Farzadfar F, Fay DF, Feigin VL, Flaxman A, Forouzanfar MH, Goto A, Green MA, Gupta R, Hafezi-Nejad N, Hankey GJ, Harewood HC, Havmoeller R, Hay S, Hernandez L, Husseini A, Idrisov BT, Ikeda N, Islami F, Jahangir E, Jassal SK, Jee SH, Jeffreys M, Jonas JB, Kabagambe EK, Khalifa SE, Kengne AP, Khader YS, Khang YH, Kim D, Kimokoti RW, Kinge JM, Kokubo Y, Kosen S, Kwan G, Lai T, Leinsalu M, Li Y, Liang X, Liu S, Logroscino G, Lotufo PA, Lu Y, Ma J, Mainoo NK, Mensah GA, Merriman TR, Mokdad AH, Moschandreas J, Naghavi M, Naheed A, Nand D, Narayan KM, Nelson EL, Neuhouser ML, Nisar MI, Ohkubo T, Oti SO, Pedroza A, Prabhakaran D, Roy N, Sampson U, Seo H, Sepanlou SG, Shibuya K, Shiri R, Shiue I, Singh GM, Singh JA, Skirbekk V, Stapelberg NJ, Sturua L, Sykes BL, Tobias M, Tran BX, Trasande L, Toyoshima H, Vasankari TJ, Veerman JL, Velasquez-Melendez G, Vlassov VV, Vollset SE, Vos T, Wang C, Wang X, Weiderpass E, Werdecker A, Wright JL, Yang YC, Yatsuya H, Yoon J, Yoon SJ, Zhao Y, Zhou M, Zhu S, Lopez AD, Murray CJ, Gakidou E (2014). Global, regional, and national prevalence of overweight and obesity in children and adults during 1980-2013: a systematic analysis for the Global Burden of Disease Study 2013. Lancet.

[2] Dali-Youcef N, Mecili M, Ricci R, Andres E (2013). Metabolic inflammation: connecting obesity and insulin resistance. Ann Med.

[3] Moreno-Navarrete JM, Sabater M, Ortega F, Ricart W, Fernandez-Real JM (2012). Circulating zonulin, a marker of intestinal permeability, is increased in association with obesity-associated insulin resistance. PLoS One.

[4] Pacifico L, Bonci E, Marandola L, Romaggioli S, Bascetta S, Chiesa C (2014). Increased circulating zonulin in children with biopsy-proven nonalcoholic fatty liver disease. World J Gastroenterol.

[5] Zhang D, Zhang L, Zheng Y, Yue F, Russell RD, Zeng Y (2014). Circulating zonulin levels in newly diagnosed Chinese type 2 diabetes patients. Diabetes Res Clin Pract.

[6] Friedman JM, Halaas JL (1998). Leptin and the regulation of body weight in mammals. Nature.

[7] Considine RV, Sinha MK, Heiman ML, Kriauciunas A, Stephens TW, Nyce MR, Ohannesian JP, Marco CC, McKee LJ, Bauer TL, et al (1996). Serum immunoreactive-leptin concentrations in normal-weight and obese humans. N Engl J Med.

[8] Kuczmarski RJ, Ogden CL, Guo SS, Grummer-Strawn LM, Flegal KM, Mei Z, Wei R, Curtin LR, Roche AF, Johnson CL (2002). 2000 CDC Growth Charts for the United States: methods and development. Vital Health Stat 11.

[9] Tanner JM, Whitehouse RH (1976). Clinical longitudinal standards for height, weight, height velocity, weight velocity, and stages of puberty. Arch Dis Child.

[10] Rosner B, Prineas RJ, Loggie JM, Daniels SR (1993). Blood pressure nomograms for children and adolescents, by height, sex, and age, in the United States. J Pediatr.

[11] Valerio G, Licenziati MR, Iannuzzi A, Franzese A, Siani P, Riccardi G, Rubba P (2006). Insulin resistance and impaired glucose tolerance in obese children and adolescents from Southern Italy. Nutr Metab Cardiovasc Dis.

[12] Cani PD, Bibiloni R, Knauf C, Waget A, Neyrinck AM, Delzenne NM, Burcelin R (2008). Changes in gut microbiota control metabolic endotoxemia-induced inflammation in high-fat diet-induced obesity and diabetes in mice. Diabetes.

[13] Cani PD, Possemiers S, Guiot Y, Everard A, Rottier O, Geurts L, Naslain D, Neyrinck A, Lambert DM, Muccioli GG, Delzenne NM (2009). Changes in gut microbiota control inflammation in obese mice through a mechanism involving GLP-2-driven improvement of gut permeability. Gut.

[14] Ferraris RP, Casirola DM, Vinnakota RR (1993). Dietary carbohydrate enhances intestinal sugar transport in diabetic mice. Diabetes.

[15] Ferraris RP, Vinnakota RR (1995). Intestinal nutrient transport in genetically obese mice. Am J Clin Nutr.

[16] Gummesson A, Carlsson LM, Storlien LH, Backhed F, Lundin P, Löfgren L, Stenlöf K, Lam YY, Fagerberg B, Carlsson B (2011). Intestinal permeability is associated with visceral adiposity in healthy women. Obesity (Silver Spring).

[17] Wapenaar MC, Monsuur AJ, Bodegraven AA, Weersma RK, Bevova MR, Linskens RK, Howdle P, Holmes G, Mulder CJ, Dijkstra G, Heel DA, Wijmenga C (2008). Associations with tight junction genes PARD3 and MAGI2 in Dutch patients point to a common barrier defect for coeliac disease and ulcerative colitis. Gut.

[18] Fasano A (2000). Regulation of intercellular tight junctions by zonula occludens toxin and its eukaryotic analogue zonulin. Ann N Y Acad Sci.

[19] Wang W, Uzzau S, Goldblum SE, Fasano A (2000). Human zonulin, a potential modulator of intestinal tight junctions. J Cell Sci.

[20] Backhed F, Ley RE, Sonnenburg JL, Peterson DA, Gordon JI (2005). Host-bacterial mutualism in the human intestine. Science.

[21] La Serre CB, Ellis CL, Lee J, Hartman AL, Rutledge JC, Raybould HE (2010). Propensity to high-fat diet-induced obesity in rats is associated with changes in the gut microbiota and gut inflammation. Am J Physiol Gastrointest Liver Physiol.

[22] El Asmar R, Panigrahi P, Bamford P, Berti I, Not T, Coppa GV, Catassi C, Fasano A (2002). Host-dependent zonulin secretion causes the impairment of the small intestine barrier function after bacterial exposure. Gastroenterology.

[23] Fasano A (2011). Zonulin and its regulation of intestinal barrier function: the biological door to inflammation, autoimmunity, and cancer. Physiol Rev.

[24] Lammers KM, Lu R, Brownley J, Lu B, Gerard C, Thomas K, Rallabhandi P, Shea-Donohue T, Tamiz A, Alkan S, Netzel-Arnett S, Antalis T, Vogel SN, Fasano A (2008). Gliadin induces an increase in intestinal permeability and zonulin release by binding to the chemokine receptor CXCR3. Gastroenterology.

[25] Deng Y, Scherer PE (2010). Adipokines as novel biomarkers and regulators of the metabolic syndrome. Ann N Y Acad Sci.

[26] Brock M, Trenkmann M, Gay RE, Gay S, Speich R, Huber LC (2011). MicroRNA-18a enhances the interleukin-6-mediated production of the acute-phase proteins fibrinogen and haptoglobin in human hepatocytes. J Biol Chem.

[27] Zak-Golab A, Kocelak P, Aptekorz M, Zientara M, Juszczyk L, Martirosian G, Chudek J, Olszanecka-Glinianowicz M (2013). Gut microbiota, microinflammation, metabolic profile, and zonulin concentration in obese and normal weight subjects. Int J Endocrinol.

[28] Goff DC, D’Agostino RB, Haffner SM, Otvos JD (2005). Insulin resistance and adiposity influence lipoprotein size and subclass concentrations. Results from the Insulin Resistance Atherosclerosis Study. Metabolism.

[29] Catli G, Anik A, Tuhan HÜ, Kume T, Bober E, Abaci A (2014). The relation of leptin and soluble leptin receptor levels with metabolic and clinical parameters in obese and healthy children. Peptides.

[30] Matthews DR, Hosker JP, Rudenski AS, Naylor BA, Treacher DF, Turner RC (1985). Homeostasis model assessment: insulin resistance and beta-cell function from fasting plasma glucose and insulin concentrations in man. Diabetologia.

[31] Fasano A (2009). Surprises from celiac disease. Sci Am.

