# Correction

**DOI:** 10.4274/jcrpe.galenos.2020.e002

**Published:** 2020-09-02

**DOI: 10.4274/jcrpe.3682**

Küme T, Acar S, Tuhan H, Çatlı G, Anık A, Gürsoy Çalan Ö, Böber E, Abacı A. The Relationship between Serum Zonulin Level and Clinical and Laboratory Parameters of Childhood Obesity. *J Clin Res Pediatr Endocrinol* 2017;9:31-38.

The mistake has been made inadvertently. “Financial Disclosure: The authors declared that this study received no financial support.” has been corrected as “Financial Disclosure: This study was funded by Dokuz Eylul University Department of Scientific Research Projects (No: 2017.KB.SAĞ.013)”

